# Intraepithelial lymphocyte distribution differs between the bulb and the second part of duodenum

**DOI:** 10.1186/1471-230X-13-111

**Published:** 2013-07-10

**Authors:** Olga Bednarska, Simone Ignatova, Charlotte Dahle, Magnus Ström

**Affiliations:** 1 Department of internal medicine, Oskarshamn hospital, Oskarshamn, Sweden; 2 Department of pathology, Linköping university hospital, Linköping, Sweden; 3 Department of clinical immunology, Linköping university hospital, Linköping, Sweden; 4 Department of Gastroenterology and Hepatology, Linköping university hospital, Linköping, Sweden; 5 Department of Clinical and Experimental Medicine, Faculty of Health Sciences, Linköping University, Linköping, Sweden

## Abstract

**Background:**

Evaluation of intraepithelial duodenal lymphocytosis (IDL) is important in celiac disease (CD). There is no established cut-off value for increased number of IELs in the bulb.

We therefore investigated the relation between IEL counts in the bulb and duodenal specimens in non-celiac subjects.

**Methods:**

The number of CD3+ IELs was determined in specimens from the second part of the duodenum and from the bulb in 34 non-celiac subjects. The numbers of IELs in the villus tip and sides were counted and the quotient tip/side was calculated. HLA DQ2/DQ8 and serum antibodies against transglutaminase were analysed.

**Results:**

The mean number of IELs per 100 enterocytes (95% CI) in specimens was 14.7 (11.8-17.6) in the bulb, and 21.2 (17.0-25.5) in the second part of the duodenum (p<0.01). There was no difference in IEL count or distribution comparing patients carrying or lacking HLA DQ2/DQ8.

**Conclusions:**

IEL count in non-celiac, HLA DQ2/DQ8 positive or negative patients is significantly lower in the bulb than in the second part of the duodenum. These findings implicate that the site of biopsy should be taken into account when considering duodenal lymphocytosis.

## Background

Intraepithelial lymphocytes (IELs) constitute a distinct population of T cells in the basal portion of the gut epithelium. Intraepithelial lymphocytosis is one of the criteria for the diagnosis of celiac disease (CD) [1]. However, it is an unspecific finding that can be found in many other inflammatory conditions with or without concomitant infection [2,3] and can also be drug-induced [4]. In western countries, intraepithelial lymphocytosis is associated with Helicobacter pylori (HP) infection provided that CD has been excluded [5,6]. In patients with HP-infection high numbers of IELs in the duodenum [7] decrease after HP-eradication [5].

Intraepithelial lymphocytosis was recently defined as a normal villous architecture with >25 IELs per 100 enterocytes in the duodenal mucosa, as judged by immunohistochemistry [8]. Increased numbers of IELs has been considered to be of high diagnostic value in CD, provided the distribution is even along the villus [4,9,10] or if the IELs predominantly have an apical localisation [4,11,12] In recent years, it has been claimed that biopsies from the bulb are essential when considering CD [3,13,14], and this is now recommended in addition to biopsies from the second part of the duodenum in all patients who are investigated for CD [14-16]. A reference value for IELs in the bulb has not been settled, but it has been assumed to be in the same range as in the second part of duodenum [6,17].

The aims of this study were to assess (i) the numbers and distribution of IELs in biopsies from the bulb and the second part of the duodenum in subjects without HP (ii) whether there are any differences in numbers or distribution of IELs between subjects with or without HLA DQ2/8.

## Methods

### Study population

Patients successfully treated for non-ulcerating HP gastritis ten years ago were recruited to minimise any influence of recent HP-infection. A prerequisite was that they had a negative urea breath test 6 weeks after HP eradication and, at control gastroscopy one year later, a negative rapid urease test and no HP present at histological examination. Further, the study subjects (n=57) were selected on the basis of available frozen (−70°C) serum samples (S1), which were analyzed for antibodies against tissue transglutaminase (anti-tTG) to exclude CD with high probability. The patients were asked to participate in a follow-up control with endoscopy. Out of the 57 identified subjects 23 declined or were disqualified from a new endoscopy due to high age.

The remaining 34 subjects consisted of 14 men and 20 women with a mean age of 66 years (range 37–88). No patients suffered from any disease known to be associated with increased IEL except seven subjects who had regular intake of non-steroidal anti-inflammatory drugs. All patients underwent blood sampling (S2).

### Esophago-duodenoscopy

At the upper gastrointestinal endoscopy two biopsy specimens were taken from the bulb (in all but one case) and two specimens from the second part of the duodenum below the papilla major. Biopsies for rapid urease test (Pronto Dry®, Gastrex, Brignais, France) were taken from both antrum and corpus [18].

### Histopathology

Biopsy specimens from duodenum and the bulb were fixed in formalin. Paraffin blocks were cut in 4-μm sections and stained with hematoxyline-eosine (HE) to assess the villous/crypt ratio and immuno-histochemical staining (IHC) with anti-CD3 monoclonal antibody (monoclonal IgG1 mouse anti-human CD3 antibodies, Clone F7.2.38. Dako, Glosrup, Denmark) to visualise IELs. The specimens were independently examined by an experienced pathologist (SI) and a resident (OB). In each specimen areas of mostly expressed histopathological changes including low villus/crypt ratio, increased numbers of lymphocytes were recognized. Lymphocytes were estimated in CD3 immunostained sections counting 500 enterocytes preferably in five adjacent, well oriented villi. In each of the five villi the number of IELs per 20 enterocytes in the tip of the villus and per 80 enterocytes in the side of the villus (altogether 100 enterocytes per villus) was calculated. An upper limit of 25 lymphocytes per 100 enterocytes and a minimal villus/crypt (V:C) ratio of 3:1 in the second part of the duodenum were considered normal [19]. A value of top-IELs/100 enterocytes (five villi) and side IELs/400 enterocytes (five villi) converted to IELs number/100 enterocytes was tabulated. Furthermore, IELs tip/side score (T/S) was calculated in order to distinguish IELs distribution pattern.

### Serum analyses

Ten year old (S1) and new (S2) serum samples were examined regarding IgA-antibodies against human recombinant tTg (Celikey, Phadia, Freiburg, Germany). The analyses were performed according to the manufacturers’ instructions and the recommended cut-off 7 AU⁄ mL was used. Total serum IgA levels were determined by a routine turbidimetric method. One patient had developed an IgA-deficiency (<0.07 g/L) in the S2 blood sampling, and in this case IgG anti-tTg was analysed.

### HLA

Typing for DQ2/DQ8 genes was performed with PCR-SSP assay (Olerup SSP®, Wien, Austria).

### Statistical analysis

Kappa statistics was used to compare histological classification between the two examiners. The study results were depicted by mean and 95% confidence interval (CI). Comparisons were performed by the t-test. P-values below 0.05 (2-sided) were considered statistically significant.

### Ethical considerations

The study was approved by the regional ethics committee (Regionala etikprövningsnämnden i Linköping), Linköping, Sweden. All subjects gave written informed consent.

## Results

### HP status

Based on a negative rapid urease test (performed during gastroenteroscopy using biopsy specimens both from the antrum and the corpus) together with negative histology for HP, a current HP infection was excluded in all but one case.

### Serology

The S1 serum samples as well as the S2 follow-up serum samples were all negative regarding anti-tTG antibodies.

### Histology

The specimen from the patient with recurrent HP infection was excluded. One patient did not have a biopsy from the bulb taken and one biopsy from the bulb was not possible to examine due to inadequate preparation.

The inter-observer results were consistent (kappa coefficient 0.67 and 0.69 respectively) regarding histopathology of the bulb (n=31) and the second part of the duodenum (n=33). The IELs values of OB were chosen for statistical analyses.

Intraepithelial lymphocytosis [8] (*i*.*e*. >25 IELs/100 enterocytes, corresponding to Marsh grade I) was found in 27% of duodenal biopsies and in 13% of bulb biopsies (p>0.05).

No biopsy revealed crypt hyperplasia. The villus:crypt-ratio was significantly lower in the bulb compared with the duodenal biopsies (mean 2.3 and 3.1 respectively, p<0.001).

The number of IELs in biopsies from the second part of the duodenum were significantly higher (p<0.01) than in biopsies from the bulb, both in the tip and the side, as well as regarding total IEL counts (Table 1). The ‘IEL distribution pattern’, IEL tip/side score, in the second part of the duodenum did not differ significantly from that in the bulb (Table 1).

**Table 1:** Intraepithelial lymphocytosis in specimens from the second part of the duodenum and from the bulb

**IELs/ 100 enterocytes**	**Tip**	**Side**	**Total**	**T/S**
Duodenum	23.0* (19.5-26.5)	21.2* (16.7-25.6)	21.6* (17.5-25.8)	1.26 (1.07-1.44)
Bulb	15.9* (12.0-19.8)	14.0* (11.1-16.9)	14.3* (11.4-17.1)	1.24 (1.06-1.43)

* p<0.01

Tip- the sum of IEL/20 enterocytes from five villi.

Side- the sum of IEL/80 enterocytes from five villi divided by four.

Total- the sum of five villi’s IEL/20 enterocytes in the tip + IEL/80 enterocytes in the side divided by five.

T/S the number of tip enterocytes divided by the number of side enterocytes.

Mean values and 95% CI.

Subjects regularly using NSAID (n=7) had a tendency (p=0.07) of a higher IEL count in the second part of the duodenum mean 29.1 (95% CI, 11.9-46.2) compared with subjects not using NSAID 19.6 (95% CI, 16.1-23.1).

### HLA genotyping

The HLA DQ2 and/or DQ8 were found in 41%.

IEL counts did not differ between patients with or without HLA DQ2/8 (Table 2).

**Table 2:** Number of IELs per 100 enterocytes in the second part of the duodenum and in the bulb in patients with and without HLADQ2/8

**IELs/100 enterocytes**	**Tip**	**Side**	**Total**	**T/S**
DQ2/8 duodenum	22.1 (14.9-29.2)	21.5 (11.1-31.9)	21.8 (12.2-31.5)	1.27 (0.90-1.63)
Non-DQ2/8 duodenum	23.6 (19.6-27.6)	20.9 (16.7-25.2)	21.5 (17.5-25.4)	1.25 (1.03-1.46)
DQ2/8 bulb	16.8 (7.9-25.7)	14.0 (9.0-19.0)	14.2 (8.8-19.6)	1.28 (0.92-1.64)
Non-DQ2/8 bulb	15.3 (11.9-18.6)	14.0 (10.0-17.9)	14.3 (10.7-17.9)	1.22 (0.99-1.45)

Tip- the sum of IEL/20 enterocytes from five villi.

Side- the sum of IEL/80 enterocytes from five villi divided by four.

Total- the sum of five villi’s IEL/20 enterocytes in the tip + IEL/80 enterocytes in the side divided by five.

T/S the number of tip enterocytes divided by the number of side enterocytes.

Mean values and 95% CI.

## Discussion

The importance of taking biopsies from the bulb in the diagnostic workup of celiac disease has been reported lately [13-16,20]. There is no established cut-off value for increased number of IELs in the bulb. Whether or not intraepithelial lymphocytosis is restricted to the bulb in early stages of CD is not known. Most publications in this field evaluate the number of IELs in the second part of the duodenum [19], and these results have automatically been considered to apply also for the bulb.

We found IEL counts to be significantly lower in the bulb compared to the second part of the duodenum. There are a few previous reports on the number of IELs in the bulb compared with the second part of the duodenum in children and adults without CD, and in these studies no significant differences were found [6,13,17,21]. The lower IEL counts in the bulb reported here might be of importance when defining intraepithelial lymphocytosis in specimens from this area, as the accepted cut-off in the second part of the duodenum *i*.*e*. >25 IELs per 100 enterocytes may be too high for the bulb. Our findings indicate that different cut-off levels should be applied in the bulb compared with the distal duodenum, to define intraepithelial lymphocytosis at these sites. This may be important in order not to underestimate intraepithelial lymphocytosis in cases where duodenal biopsies are only available from the bulb.

HLA DQ2 and DQ8 are closely linked to CD [22], but seems also to be overrepresented in HP infection [23]. Hence, the high occurrence of these HLA alleles among our subjects with previous HP-infection was expected. In this study, IEL counts did not differ between patients with or without DQ2/8. In order to obtain reference values for IELs in true non-CD, biopsies from the second part of duodenum have recently been explored in DQ2/8-negative controls [24]. The optimal approach might be to define IEL cut-off levels based on biopsies from healthy DQ2/8-positive persons. To reassure that our patients neither had latent or silent CD nor an active or recent HP infection, we selected cases who had no clinical signs of gluten intolerance and negative anti-tTg antibody tests also at follow-up after a ten year observation period., As IEL counts were the same in cases with or without DQ2/DQ8, it seems justified to apply the same reference value regardless of HLA DQ2/DQ8 status.

The microscopic examination of biopsy specimens from both the bulb and the second part of the duodenum, overall revealed an even IEL distribution along the villus, but with a tendency of increased IEL counts apically. We performed an exact enumeration of IELs, in contrast to the observer-dependent estimation which has been applied so far [25]. Until now, only one study has reported a duodenal IEL ratio by dividing IEL counts in the villus tip with IEL counts at the side of the villus [12]. The results of the present study argue against the prevailing notion that an apical predominance or ’even-along-the-villus’ pattern is a finding suggestive of CD [4,9,10,26], since we found that this was common also among non-CD subjects.

A major drawback of this study is the limited study population. However, this was due to our wish to exclude CD cases by a 10-year follow-up of subjects testing negative with regard to celiac-specific antibodies. Therefore, identification of stored serum samples was crucial for the study but at the same time a limiting condition, especially as we also wished to include patients with HLA DQ2/8 *i*.*e*. those with a genetic prerequisite to develop CD [22]. Despite the limitation, this long observation time is at the same time a major advantage of our study. Another study virtue is the high kappa coefficient value of the IEL counts reported by the two independent investigators, reassuring the reliability of our results.

## Conclusion

In this study we found (i) lower number of IELs in the bulb compared with the second part of the duodenum in subjects without any sign of CD. The distribution of IELs was the same in bulb and the second part of the duodenum. (ii) There was no difference in number or distribution of IELs between subjects regardless of HLA-DQ2 or -DQ8 occurrence.

Our findings implicate that the approach to evaluate duodenal lymphocytosis needs deeper appreciation with regard to whether specimens from the bulb or the descending part of the duodenum are examined.

## Competing interests

The authors declare that they have no competing of interest.

## Authors’ contributions

OB contributed to conception and design, acquisition, analysis and interpretation of data and wrote the first draft of the manuscript. SI and LD contributed to acquisition, analysis and interpretation of data. MS contributed to conception and design, acquisition, analysis and interpretation of data and finished the manuscript. All authors critically read and approved the final version.

## Pre-publication history

The pre-publication history for this paper can be accessed here:

http://www.biomedcentral.com/1471-230X/13/111/prepub
